# 273. Neurological Involvement Caused by Intracellular Bacteria

**DOI:** 10.1093/ofid/ofab466.475

**Published:** 2021-12-04

**Authors:** Fatma Hammami, Makram Koubaa, Amal Chakroun, Khaoula Rekik, Chakib Marrakchi, Fatma Smaoui, Mounir Ben Jemaa

**Affiliations:** Infectious Diseases Department, Hedi Chaker University Hospital, University of Sfax, Tunisia, Sfax, Sfax, Tunisia

## Abstract

**Background:**

Infection of the central nervous system is a severe and fatal disease. Causative agents include bacteria, viruses or fungi. Intracellular bacteria are not only overlooked, but also underdiagnosed. We aimed to study the clinical, laboratory and evolutionary features of neurological involvement caused by intracellular bacteria.

**Methods:**

We conducted a retrospective study including all patients hospitalized in the infectious disease department for neurological involvement caused by intracellular bacteria between 1995 and 2020. The diagnosis was confirmed by serology.

**Results:**

We encountered 76 cases among which 43 were males (56.6%). The mean age was 32±18 years. The revealing symptoms included fever (97.4%), cephalalgia (73.7%), vomiting (64.5%) and arthralgia (51.3%). Lumbar puncture revealed a median white blood cell count of 120[56-340]/mm^3^. Lymphocytic pleocytosis was noted in 62% of the cases. Elevated cerebrospinal fluid (CSF) protein level was noted in 37 cases (48.7%) with a median of 0.84[0.6-1.37] g/L. Low CSF fluid glucose level was noted in 14 cases (18.4%). There were 70 cases (92.1%) of meningitis and 6 cases of meningoencephalitis (7.9%). The causative agent included *Rickettsia* species in 47 cases (61.8%), *Brucella* species in 17 cases (22.4%) and *Mycoplasma* species in 12 cases (15.8%). Laboratory investigations included elevated C-reactive protein levels (40.7%), thrombocytopenia (32.8%) and increase in hepatic enzyme levels (21%). Anemia was noted in 27 cases (35.5%), leukocytosis in 24 cases (31.5%) and leucopoenia in 6 cases (7.8%). Blood and CSF cultures were positive for *Brucella* in 2 cases (2.6%) and 5 cases (6.5%), respectively. The mean duration of treatment was 156±94 days for brucellosis cases, 9±4 days for rickettsiosis cases and 10±6 days for *Mycoplasma* cases. The disease evolution was favorable in 72 cases (94.7%). Four patients were dead (5.3%). Complications were noted in 5 cases (6.5%) and sequelae in 2 cases (2.6%).

**Conclusion:**

Intracellular bacteria including *Brucella*, *Rickettsia* and *Mycoplasma* species should be considered in front of neurological symptoms. Meningitis with lymphocytic pleocytosis was the most common clinical presentation. An early diagnosis followed by the adequate treatment might avoid complications and death.

**Disclosures:**

**All Authors**: No reported disclosures

